# 非小细胞肺癌的免疫治疗：改善患者预后的新方法

**DOI:** 10.3779/j.issn.1009-3419.2013.04.09

**Published:** 2013-04-20

**Authors:** Frances A. Shepherd, Jean-Yves Douillard, George R. Blumenschein, 玲 张

**Affiliations:** 1 Department of Medical Oncology and Hematology, University Health Network, Princess Margaret Hospital and the University of Toronto, Toronto, Ontario, Canada; 2 Department of Medical Oncology, ICO R Gauducheau, St-Herblain France; 3 Department of Toracic/Head and Neck Medical Oncology, Te University of Texas M. D. Anderson Cancer Center, Houston, Texas; 4 同济大学附属上海市肺科医院肿瘤科

**Keywords:** 非小细胞肺癌, 免疫治疗, 疫苗, 多克隆抗体, 免疫调节

## Abstract

**简介:**

通常，非小细胞肺癌（non-small cell lung cancer, NSCLC）诊断已为晚期，且预后较差。目前的NSCLC标准治疗总体治愈率低，有必要开发新的治疗方法。我们在本综述中提供了最新的免疫治疗干预临床数据，该手段可能能够提高免疫系统对细胞的应答。

**方法:**

我们针对临床应用免疫疗法治疗NSCLC，检索了PubMed上的文章以及最近肿瘤学术会议上的摘要。

**结果:**

Ⅱ期临床研究结果表明，靶向肿瘤细胞本身或其异常表达的肿瘤标志物的疫苗治疗（mucin1，黑色素瘤相关抗原3，或表皮生长因子），有望作为NSCLC免疫疗法。非抗原免疫治疗，如抗细胞毒T淋巴细胞抗原4单克隆抗体、talactoferrin alfa和toll-样受体9拮抗剂，作用于激活的免疫系统，与肿瘤抗原无关，可用于晚期NSCLC的治疗。目前一些免疫治疗正在进行Ⅲ期研究，以确定最佳治疗方案，并与NSCLC标准治疗对照，确定其临床疗效。

**结论:**

越来越多的证据表明肺部肿瘤存在免疫应答。免疫治疗，包括疫苗治疗和非抗原免疫调节方法，可改善NSCLC的预后。而且，提高抗肿瘤免疫应答的治疗，与化疗有协同作用。生物标志物的明确以及免疫治疗作用机制的进一步阐明对于确定哪些患者更可能从免疫治疗中获益至关重要。

世界范围内，肺癌是男性癌症死亡的首要原因，对女性则仅次于乳腺癌^[1]^。在美国，支气管和肺癌约占癌症死亡的30%^[2]^。肺癌是一种异质性疾病，包括两种主要的组织学亚型：非小细胞肺癌(nonsmall cell lung cancer, NSCLC)和小细胞肺癌(small cell lung cancer, SCLC)。NSCLC约占所有病例的85%，包括几种亚型^[3, 4]^。绝大多数NSCLC患者诊断时即不可手术、局部晚期或发生转移，而其它在疾病较早阶段得到诊断的患者则出现复发并最终死于该疾病。

传统的晚期NSCLC治疗为4-6周期含铂两药化疗，进展后进行积极的二线治疗^[3, 5]^。一线化疗后无进展患者采用的维持治疗最近也成为了某些患者新的治疗模式^[6-8]^。

在一线化疗中加入贝伐珠单抗或西妥昔单抗等靶向药物，能够使疗效得到某些改善^[9-11]^。但是，鳞癌患者因为出血性并发症的风险增高而不适于接受贝伐珠单抗治疗，另外由于疗效有限也不适于接受培美曲塞治疗^[12]^。携带活化表皮生长因子受体(epidermal growth factor receptor, EGFR)突变的患者可从最初的EGFR酪氨酸激酶抑制剂(tyrosine kinase inhibitors, TKIs)(吉非替尼或厄洛替尼)治疗中获益^[13, 14]^。与鳞癌相比，这些突变更常见于腺癌，且与非吸烟者及东亚裔有关^[15]^。

过去的十年中，尽管引进了新的化疗药以及若干分子靶向药物，患者的预后依然很差，总体治愈率不足20%。很明显，需要开发新的治疗方法。本文提供了NSCLC抗肿瘤免疫应答的概述，并回顾了几种免疫治疗可获得的临床数据，重点关注目前Ⅲ期临床研究中的治疗方法。

## 肺癌中的抗肿瘤免疫应答

当肿瘤细胞碎片被抗原提呈细胞(antigen-presenting cells, APCs)(尤其是树突细胞)内化、加工，并与Ⅰ型和Ⅱ型主要组织相容性复合物(major histocompatibility complex, MHC)结合出现于APC胞外表面时，免疫系统可产生抗肿瘤应答^[16]^。当引流至邻近的淋巴结并成熟后，这些APCs可与幼稚T淋巴细胞相互作用，触发肿瘤特异性CD4^+^辅助分子和CD8^+^细胞毒性T细胞的活化与增殖(图 1) ^[17]^。T细胞的活化需要APCs上的抗原-MHC复合体和幼稚T细胞表面的T细胞受体相互作用，以及APCs上B7.1(CD80)或B7.2(CD86)与T细胞上CD28共刺激的相互作用。若不能充分激活该共刺激通路，则产生免疫耐受性^[16, 18]^。

**1:**
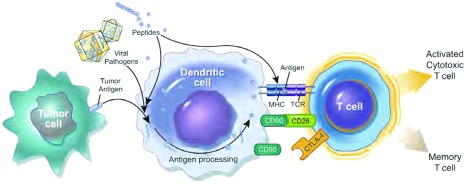
树突状细胞-T细胞相互作用产生抗肿瘤的细胞毒性T细胞^[17]^。CTLA-4，细胞毒性T淋巴细胞抗原-4；MHC，主要组织相容性复合体; TCR，T细胞受体。

T细胞活化后，细胞毒性T淋巴细胞抗原- 4 (CTLA-4)在T细胞表面表达^[19, 20]^。CTLA-4与CD80/CD86发生高亲和力结合，并给出抑制性信号限制T细胞的进一步活化。该机制有助于维持对正常细胞表面宿主抗原的耐受性，可预防淋巴增殖性疾病。但是，肿瘤可通过诱导耐受性或产生T细胞介导破坏的抗性，而逃逸免疫系统^[21]^。

肿瘤细胞可能能够高表达CD4^+^CD25^+^调节性T细胞，该细胞可抑制肿瘤特异性CD4^+^和CD8^+^效应细胞的功能与增殖^[22, 23]^。骨髓来源的抑制细胞和肿瘤相关巨噬细胞的增多也可抑制T细胞增殖及其效应子功能，并促进肿瘤的生长和转移^[24, 25]^。

除此之外，肿瘤可通过促进或抑制一系列因子的表达从而阻断抗肿瘤免疫细胞的活化、增殖或功能^[26]^。例如，肿瘤抗原的下调或MHC-I类分子的表达，以及改变免疫调节细胞因子的分泌^[16]^。

尽管肺癌不是典型的“免疫原性”恶性肿瘤，但越来越多的证据表明肺部肿瘤可能存在免疫应答，其强度与患者的预后相关。肺癌患者肿瘤标本的回顾性分析表明，抗肿瘤细胞的免疫应答与预后呈正相关。几项临床研究表明，较高的CD4^+^和/或CD8^+^T细胞肿瘤内浸润程度与更长的早期NSCLC的生存期有关^[27-29]^。在最大的研究中，335例手术切除的Ⅰ期到ⅢA期NSCLC患者中，基质CD8^+^和CD4^+^T细胞计数高与疾病特异性高存活率独立相关^[28]^。另一组患者中，CD4^+^和CD8^+^T细胞的同时高度浸润是独立的预后因素，提示CD4^+^和CD8^+^细胞协同作用可产生比各自单独作用更强的免疫应答^[27]^。癌巢中CD8^+^T细胞高度浸润与鳞癌有关，而CD4^+^T细胞浸润与组织学无关。74例早期NSCLC中，B细胞滤泡旁存在有成熟的树突状细胞和T细胞簇，含这些细胞簇的三级淋巴结构的密度与总体生存期(overall survival, OS)、疾病特异性OS、无瘤生存期密切相关^[30]^。肿瘤内树突状细胞少，则肿瘤浸润淋巴细胞的密度也低。与肿瘤周围的基质组织相比，较多的癌巢中肿瘤浸润巨噬细胞和CD8^+^T细胞数量，与Ⅳ期NSCLC患者预后较好独立相关^[31]^。

如上所述，CD4^+^CD25^+^调节性T细胞可抑制抗肿瘤免疫。几项回顾性研究提示肿瘤浸润调节性T细胞的高表达与早期NSCLC疾病复发相关^[32, 33]^。在一组手术切除的Ⅰ期NSCLC患者中，高调节性T细胞与肿瘤浸润T细胞的比例与疾病复发相关^[32]^。

该证据支持如下假说，通过免疫治疗来诱导或强化免疫应答可作为肺癌的一种治疗方法，包括内科治疗远远不够的患者亚群。免疫治疗的目的是强化免疫系统对肺癌细胞的应答。例如，免疫治疗制剂的作用机制可能促进更多的免疫介导的细胞毒效应器机制的产生和/或可能削弱促进肿瘤细胞免疫耐受性的调节机制。疫苗治疗和非抗原免疫治疗是目前正在研发的肺癌治疗方法。

## 疫苗治疗

肺癌治疗中，几种不同的疫苗治疗策略已得到评估(更详尽的综述请参见Bradbury和Shepherd的文章) ^[34]^。大部分数据是根据抗原特异性疫苗和肿瘤细胞疫苗的使用给出的，树突细胞疫苗仅有初步研究数据(表 1) ^[16, 35-40]^。一种称为佐剂的非特异免疫刺激因子与疫苗联合使用，以促使APCs运动到免疫部位，并促进APCs摄取疫苗抗原^[16, 34]^。

**1:** 疫苗的Ⅱ期临床研究结果，目前正在进行Ⅲ期临床研究

疫苗类型	名称	靶点	研究设计	结果	参考文献
抗原特异性	L-BLP25	MUC1	171例ⅢB/Ⅳ期NSCLC患者，接受L-BLP25或最佳支持治疗	L-BLP25治疗的ⅢB期患者生存期改善(分别为30.6个月和13.3个月，*P*=0.16)	35, 41
抗原特异性	TG4010	MUC1(也包括IL-2)	148例初治ⅢB/Ⅳ期NSCLC患者接受吉西他滨/顺铂土TG4010	TG4010+吉西他滨/顺铂改善6个月时的PFS率(分别为44%和35%, *P*=0.03)；基线NK细胞正常水平的患者TG4010+吉西他滨/顺铂改善OS(分别为17.1个月和11.3个月)	16, 36, 44, 45
抗原特异性	MAGE-A3	MAGE-A3	182例完全切除的ⅠB/Ⅱ期、MAGE-A3阳性NSCLC患者2:1比例接受MAGE-A3疫苗或安慰剂	与安慰剂相比，有延长无病间期的趋势(HR=0.74; *P*=0.107)；含有与高复发风险相关基因表达特征的患者疗效更佳	37
抗原特异性	CimaVax EGF	EGF	80例复治NSCLC患者1:1接受疫苗或安慰剂治疗	抗体应答与血清EGF下降直接相关；与未免疫患者相比，免疫的患者生存期延长；60岁以下患者差异具有统计学意义(中位OS分别为11.47个月和5.33个月，*P*=0.012, 4)	38
肿瘤细胞	Belagenpumatucel-L	四个肺癌细胞株+TGF*β*2	75例NSCLC患者接受1.25, 2.5或5×10^7^/剂	晚期患者缓解率达15%；与低剂量组相比，两个高剂量组的2年生存率更长(分别为52%和20%)；每月接受2.5×10^7^/剂的21例患者的OS为18.5个月	39, 40
NSCLC，非小细胞肺癌；EGF，表皮生长因子；HR，风险比；IL-2，白介素-2；L-BLP25，脂质BLP25；MUC1，粘液素1；NK，自然杀伤细胞；OS，总体生存期； PFS，无进展生存期；TGF，转化生长因子。 注：本表得到版权所有者© International Association for the Study of Lung Cancer复制许可。					

### 抗原特异性疫苗

可应用于疫苗治疗的抗原应具有肿瘤特异性表达模式(即，肿瘤中唯一的或异常的表达)，在该类肿瘤中普遍存在，在疾病进展过程中持续存在(即，早期和转移性疾病中均有表达)。理想状况是，存在某些证据表明它能够促进抗肿瘤免疫应答^[34]^。

脂质体BLP25疫苗(L-BLP25)，Stimuvax(德国默克，达姆施塔特，德国)，以抗原mucin1(MUC1)的暴露的核心肽为靶点，该肽在多种恶性肿瘤中过表达^[41]^，其中包括约86%的腺癌和74%的其它类型NSCLCs ^[42]^。MUC1通常表达于分泌粘液的表皮细胞顶面，但在肿瘤细胞中则出现异常的糖基化模式。肿瘤细胞中MUC1的表达与凋亡受抑、免疫应答抑制、化疗耐药性以及不良的预后有关。L-BLP25疫苗由脂质构成，易于被APCs摄取并促使其将疫苗输送给免疫细胞。

针对171例一线治疗后未进展的ⅢB/Ⅳ期NSCLC患者进行了一项Ⅱ期随机研究以评估L-BLP25^[35]^。患者随机接受L-BLP25联合最佳支持治疗(best supportive care, BSC)或单独的BSC。临床前模型证实L-BLP25组患者接受单次低剂量环磷酰胺300 mg/m2静脉推注(Ⅳ)以提高免疫治疗疗效，之后每周一次皮下注射1, 000 µg疫苗进行免疫，共8次，接着每6周一次维持免疫。疫苗治疗组的OS较好(分别为17.4个月和13.0个月)，但治疗组间的差异无统计学意义[风险比(hazard ratio, HR) =0.739；*P*=0.112]，这可能是样本量导致的。但是，之后的分析表明仅65例ⅢB期患者获益于L -BLP25。经过53个月的中期随访后，与单独BSC相比，L -BLP25能够持续有效地改善ⅢB期患者生存(分别为30.6个月和13.3个月；*P*= 0.16) ^[41]^。

在这些研究发现的基础上，启动了一项国际多中心、随机、Ⅲ期临床研究START(激活的NSCLC靶向抗原应答)，比较连续或同步放化疗后未进展的不可手术Ⅲ期NSCLC患者接受安慰剂和L-BLP25疫苗治疗的疗效(clinicaltrials.gov编号NCT00409188)。计划入组1, 476例，OS为主要终点。2011年6月结束入组筛选。

TG4010，另一种以MUC1为靶点的疫苗，是一种基于表达完整MUC1序列和白介素-2(interleukin-2, IL-2)修饰后Ankara病毒的重组病毒载体^[43]^。诱导IL-2以刺激T细胞应答，所以，该疫苗可能能够激活或强化直接作用于表达MUC1肿瘤细胞的细胞应答。

一项针对148例之前未经治疗的ⅢB/Ⅳ期NSCLC患者的随机IIb期临床研究对TG4010进行了评估^[16, 36]^。随机分配患者接受一线顺铂/吉西他滨化疗，联合或不联合TG4010。最初6周每周一次皮下注射该疫苗，之后每3周一次，直到疾病进展。与单纯的化疗相比，联合TG4010能够明显改善主要研究终点6个月时的无进展生存(progression-free survival, PFS)率(分别为44%和35%；*P*=0.03)。两组的中位OS无显著差异；但是对基线时自然杀伤(natural killer, NK)细胞水平正常的患者亚组(约占研究队列的75%)，联合TG4010可改善OS (分别为17.1个月和11.3个月) ^[44, 45]^。最常见的TG4010相关不良事件为注射部位反应、发热和腹痛。一项Ⅱb/Ⅲ期临床研究正在基线NK细胞活化水平正常、MUC1阳性的NSCLC患者中评估TG4010(clinicaltrials.gov编号NCT00415818)。

已经研发出一种针对表皮生长因子(epidermal growth factor, EGF)的疫苗(CimaVax EGF)，并在古巴注册上市用于治疗ⅢB/Ⅳ期成人NSCLC。自1995年以来，已经进行了5项Ⅰ/Ⅱ期和1项Ⅱ期CimaVax EGF临床研究(参见Rodriguez等的综述文章) ^[46]^。Ⅰ/Ⅱ期临床研究证实了该疫苗的免疫原性，并帮助确定了剂量、注射部位、佐剂及与化疗联合应用的可能性。Ⅱ期临床研究中，80例先前治疗过的晚期NSCLC患者按1:1随机接受该疫苗治疗，结果证实抗体应答与血清EGF下降直接相关。与未接受疫苗治疗的对照组患者相比，接受疫苗治疗的患者的生存期更长，年龄≤60岁的患者亚组出现有统计学差异(*P*=0.012, 4)(中位生存期分别为11.47个月和5.33个月)。副作用严重程度均≤2级，且发生率低于25%^[38]^。从2006年6月开始，在全国18个临床研究中心进行Ⅲ期临床研究。该研究计划入组579例晚期(ⅢB/Ⅳ期) NSCLC患者，按1:2随机分配(1例对照组对比2例治疗组)。结果按分层因素分别评估年龄>60岁亚组(*n*=381)和年龄≤60岁亚组(*n*=198)。160例患者的初步数据显示，24个月的生存率有数值差异，却无统计学意义^[46]^。此外，2010年末已开始国际多中心的Ⅲ期CimaVax EGF临床研究。

黑色素瘤相关抗原3(MAGE-A3)是另一种抗原疫苗，正在进行早期NSCLC术后辅助治疗的研究。MAGE-A3是细胞毒性T细胞识别的人类白细胞抗原A1分子上的一种肽，几乎唯一地表达于肿瘤细胞。鳞癌MAGE-A3的表达率高于腺癌，被认为可能与预后差相关^[47-50]^。

在一项国际随机Ⅱ期临床研究中，182例完全切除、MAGE-A3阳性的ⅠB/Ⅱ期NSCLC患者以2:1比例随机接受MAGE-A3疫苗或安慰剂辅助治疗。与安慰剂相比，该疫苗表现出延长无病间期的趋势(HR=0.74; *P*=0.107)，无病生存期和OS也有相似的改善，但无统计学显著性^[37]^。在携带有可能与高复发风险相关的基因特征的患者中，MAGE-A3疫苗的临床疗效(无病间期)几乎翻了一倍(HR=0.57) ^[50]^。该特征包括基线时与肿瘤微环境相关的免疫相关基因^[51]^。该方法耐受性良好，方案的依从性高^[37]^。

在这些鼓舞人心的Ⅱ期临床研究数据基础上，开展了Ⅲ期MAGRIT临床研究(MAGE-A3辅助非小细胞肺癌免疫治疗的临床研究；clinicaltrials.gov编号NCT00480025)。该研究计划在33个国家纳入2, 270例经手术切除、MAGE-A3阳性的ⅠB、Ⅱ或ⅢA期NSCLC患者，使其成为目前NSCLC辅助免疫治疗的最大临床研究^[51]^。患者以2:1比例随机接受MAGE-A3疫苗或安慰剂，在超过27个月的时间内接受13次肌内注射。无病生存期是主要研究终点，计划进行基因表达特征的确认研究。研究预计于2015年完成。该研究中将采用适用于福尔马林固定石蜡包埋标本的技术来验证该表达特征。

其它研发中的肺癌抗原特异性疫苗策略以神经节苷脂为靶点，它是一大类结构上相关的糖脂，位于细胞的外侧面，参与包括细胞-细胞识别、细胞基质连接、分化等在内的多种生物学功能。神经节苷脂是肿瘤免疫治疗的良好靶点，和匹配的正常组织相比，其在肿瘤内的含量非常高。肺癌中，首先采用抗BEC2疫苗对SCLC患者验证该方案，这是一种模拟神经节苷脂抗原GD3的抗独特型抗体。在一项小型探索性研究中，发现15例采用Bec2免疫、并与卡介苗BCG联合治疗的SCLC患者生存期延长，主要毒性为注射部位的皮肤反应，通常为轻度的，但也可非常严重^[52]^。尽管有这些令人鼓舞的初步结果，一项大型Ⅲ期临床研究却显示该治疗方法不能改善OS^[53]^。

小鼠的临床前研究数据显示，采用抗N-羟乙酸基(NGc)神经节苷脂抗独特型单克隆抗体racotumomab (过去称为1E10)进行单一治疗(参见Fernandez等的综述) ^[54]^或与化疗(低剂量环磷酰胺)联合治疗具有抗肿瘤作用^[55]^。在实体瘤中化疗免疫治疗合理的前提条件下，将该方法用于NSCLC患者。首先，进行了一项针对20例NSCLC患者的小型临床研究，应用1 mg氢氧化铝沉淀的1E10单克隆抗体，得到了两种免疫球蛋白(Ig) M和IgG亚型抗NeuGcGM3神经节苷脂强且特异性的抗体应答；该应答与中位生存期正相关^[56]^。该疫苗耐受性良好。其次，在30例晚期NSCLC患者中开始了一项随机Ⅱ期临床研究比较以racotumomab与支持性治疗，预期将在2012年9月完成(clinicaltrials.gov编号NCT01240447)。此外，自2009年1月开始了一项随机Ⅲ期临床研究，旨在比较racotumomab与支持治疗，计划接受1, 082例晚期NSCLC患者。本试验的主要终点为所有人群以及ⅢA或“干性” ⅢB期亚组患者的OS(controlled-trials.com ISRCTN47153584)。

### 肿瘤细胞疫苗

由肿瘤细胞制备的疫苗，理论上可能将患者的免疫系统暴露于大范围的肿瘤细胞抗原中^[34]^。Sipuleucel-T，是食品药品监督管理局唯一批准的治疗性肿瘤疫苗提供了这种免疫治疗概念的证明。该制剂为自体肿瘤细胞疫苗，能够明显改善去势耐受的前列腺癌患者生存期^[57]^。从实践与推理角度看，由肿瘤细胞株制备的异体疫苗优于由患者自身肿瘤细胞制备的自体疫苗。但是，异体疫苗所呈递的抗原未必是指定患者肿瘤所表达的抗原。

Belagenpumatucel-L是目前针对肺癌进行Ⅲ期临床研究的唯一一种肿瘤细胞疫苗的。该异体疫苗制备自四株肺癌细胞株，包括2株肺腺癌、1株鳞癌、1株大细胞癌，用含转化生长因子(transforming growth factor, TGF) -β2的反义转基因质粒进行转染^[39]^。设计抗TGF-β2的反义mRNA的表达以减少该细胞因子的产生，从而提高该疫苗的免疫原性。

在一项Ⅱ期临床研究中，75例NSCLC患者，包括61例治疗过的ⅢB/Ⅳ期患者，采用三个剂量中的一种(1.25、2.5、或5×10^7^细胞/剂)，每1或2个月皮下注射免疫一次，最多注射16次^[39]^。晚期患者组有15%获得了部分应答。接受高剂量治疗的两个组的生存期长于低剂量组(2年OS分别为52%和20%)。后续研究中，每月注射2.5×10^7^细胞/剂的21例Ⅳ期NSCLC患者的中位生存期为18.5个月，无显著副作用^[39, 40]^。本后续研究中，Belagenpumatucel-L治疗的的较长生存期与低循环肿瘤细胞含量相关。

这些发现推动了目前正在进行的Ⅲ期临床研究，以评估Ⅲ或Ⅳ期NSCLC患者一线化疗/化放疗后采用belagenpumatucel-L进行维持治疗的效果。患者产生完全缓解、部分缓解或疾病稳定且化疗结束后1个月仍维持稳定者随机接受belagenpumatucel-L或安慰剂，每月1次持续18个月，在第21和24个月时再次免疫。主要研究终点为OS，计划入组700例患者。预计2011年完成(clinicaltrials. gov编号NCT00676507)。

### 树突细胞疫苗(dendritic cell vaccines, DC-Vacs)

设计树突细胞疫苗用于强化肿瘤相关抗原的免疫原性。该方法中，自体树突细胞与坏死的肺癌细胞一起培养以生产得到肿瘤抗原，随后将带有抗原的树突细胞注射至皮下或者腹股沟淋巴结。初步研究显示该方法治疗NSCLC可行，且耐受性良好^[58-60]^，且正在进一步研究(clinicaltrials.gov编号NCT00103116)。最近报告了一项小规模Ⅰ期临床研究，采用电穿孔法将肿瘤裂解液注入得到新的DC-疫苗。15例不可手术的Ⅲ期或Ⅳ期NSCLC患者在2周内接受3次3、6或12×10^6^ DC-疫苗皮下注射。最大剂量DC-疫苗的耐受性良好。9例患者中有5例患者暴露肿瘤裂解液之后，疫苗使CD8^+^细胞产生更多的*γ*干扰素。而且，2例患者出现混合应答，提示具有一定的临床获益^[61]^。

总之，这些数据提示抗原特异性和肿瘤细胞疫苗是有望用于NSCLC的免疫治疗，但仍需Ⅲ期临床研究数据来确定这些干预是否改善了患者的预后，若能改善，则是哪种治疗方案。

## 非抗原免疫治疗

免疫治疗依赖于特异性抗原激发免疫应答的能力，而其它免疫治疗方法作用于已经被激活的免疫系统。因此，不论实际上是何种肿瘤抗原激活免疫应答，后面这些方法都应是有效的^[19]^。

### 抗CTLA-4单克隆抗体

设计抗CTL A-4单克隆抗体，通过阻断CTL A-4的抑制性信号以提高和延长肿瘤特异性T细胞的活化与增殖，从而产生有效的抗肿瘤免疫应答^[19, 20]^ (图 2) ^[19]^。因为这些制剂以免疫自身为靶点，它们不依赖于特异的肿瘤抗原或抗原性。进一步地，它们可能能够用于不同的肺癌亚型和分子表型，包括那些治疗需求远未满足的病例。

**2:**
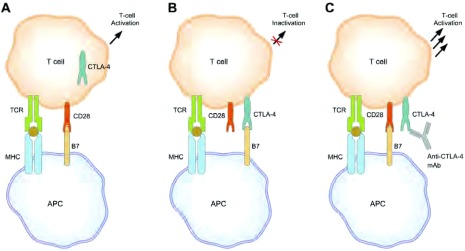
抗CTLA-4的作用机制^[19]^。APC，抗原呈递细胞；CTLA-4，细胞毒性T淋巴细胞抗原4；MHC，主要组织相容性复合体；TCR，T细胞受体。A，通过CD28连接的共刺激转导T细胞激活信号；B，CTLA-4与活化的T细胞连接下调T细胞应答；C，阻断CTLA-4的连接增强T细胞应答。

Ipilimumab是一种人化的IgG1抗CTLA-4单克隆抗体，正在评估其对肺癌患者的疗效。最近的一项Ⅲ期临床研究结果显示，与gp100单独治疗相比，伴或不伴糖蛋白100 (glycoprotein 100, gp100)肽疫苗使用ipilimumab，可改善既往接受治疗的不可手术转移性黑色素瘤患者的生存期，并伴有药物相关副反应，如腹泻、结肠炎、瘙痒、皮疹和乏力，按方案中定义的指南进行处理^[62]^。Ipilimumab在美国获准上市后，肿瘤学家可通过风险评估和减灾战略进一步获取该制剂相关的严重的免疫介导不良事件(immunemediated adverse reactions, imARs)信息^[63]^。这些发现举足轻重，因为ipilimumab是第一个统计学证明可显著改善这些患者OS的制剂，2011年3月美国食品药品监督管理局批准ipilimumab用于该适应症。

临床前证据提示化疗可补充抗CTLA-4单克隆抗体的作用，可能是通过化疗诱导细胞毒性之后的肿瘤抗原释放起作用的^[64]^。这一观察表明，联合应用抗CTLA-4单克隆抗体与化疗可改善抗肿瘤应答——与其它几项疫苗所采用的方法相一致。

通过一项随机、双盲、Ⅱ期临床研究，评估ipilimumab治疗肺癌的安全性与疗效，采用紫杉醇/卡铂联合或不联合ipilimumab治疗ⅢB/Ⅳ期NSCLC或广泛期SCLC患者^[65]^。共计204例患者随机接受下述三种治疗的一种：使用ipilimumab 10 mg/kg，前4周期同步联合紫杉醇/卡铂化疗(同步方案)，在3-6周期的紫杉醇/卡铂化疗中使用ipilimumab 10 mg/kg(分阶段组)或单纯的紫杉醇/卡铂化疗。紫杉醇/卡铂化疗6个周期后，根据最初的分组，每12周接受ipilimumab或安慰剂治疗。主要研究终点是免疫相关的PFS(immune-related PFS, irPFS)，即采用免疫相关应答标准(immune-related response criteria, irRC)评估PFS，该标准是一个开发并标准化的新应答标准，用于描述定性观察到的ipilimumab的应答模式特征(进一步解释见表 2) ^[65, 66]^。

**2:** Ipilimumab治疗晚期NSCLC^[65]^患者的疗效

	治疗组		
	Ipilimumab+紫杉醇/卡铂(同步)	Ipilimumab+紫杉醇/卡铂(分阶段)	紫杉醇/卡铂
ir^a^ PFS(月)	5.5	5.7	4.6
mWHO^b^ PFS(月)	4.1	5.1	4.2
中位OS(月)	9.7	12.2	8.3
^a^免疫相关应答标准包括评估指数病灶与可测量的新病灶^[66]^。计算基线时所有指数病灶的最大垂直径(sum of the products of the two largest perpendicular diameters, SPD)的总和，在后续肿瘤评估中计算所有指数病灶的SPD及新的可测量病灶，再求和评估肿瘤载量。根据免疫相关应答标准，完全应答的标准为间隔4周连续两次评估所有病灶消失；部分应答为间隔4周连续两次评估中与基线相比肿瘤载量下降50%；疾病稳定是指不满足部分应答标准，且肿瘤载量与最低值相比增加不超过25%。 ^b^改良的世界卫生组织定义，完全应答为所有指数/非指数病灶消失；部分应答为与基线相比，指数病灶的直径总和(sum of products of diameters, SOPD)下降50%，且无疾病进展证据；疾病进展为与研究期间记录到的最小的SOPD相比，病灶的SOPD增加25%，或任何非指数病灶的进展/出现新发病灶。完全应答或部分应答中应无新发病灶。 ir，免疫相关的；mWHO，改良的世界卫生组织；NSCLC，非小细胞肺癌；OS，总体生存期；PFS，无进展生存期。 注：本表得到版权所有者© International Association for the Study of Lung Cancer复制许可。			

晚期NSCLC治疗组的数据显示，与单纯化疗组相比，分阶段组应用ipilimumab可明显改善irPFS(分别为5.68个月和4.63个月；HR=0.686；*P*=0.026)，而同步组有改善irPFS的趋势(分别为5.52个月和4.63个月；HR=0.775；*P*=0.094) ^[65]^。若采用改良的世界卫生组织标准进行评估，分阶段组也可延长PFS(表 2)。分阶段组的中位OS为12.2个月，同步治疗组为9.69个月，单纯化疗组则为8.28个月。根据免疫相关应答标准或改良WHO标准，ipilimumab治疗组的总体缓解率均更高。

两个ipilimumab治疗组的患者接受中位4次的单克隆抗体治疗，并且倾向于接受更少周期的化疗。对照组患者紫杉醇/卡铂化疗的中位周期数为6，ipilimumab分阶段治疗组为5，而ipilimumab同步治疗组为4。Ipilimumab的安全性结果与先前单克隆抗体治疗实体瘤的研究一致，ipilimumab并不能增加紫杉醇/卡铂相关的毒性。66%接受ipilimumab的患者和55%单纯化疗的患者出现副反应，最常见的有腹泻、结肠炎、瘙痒、皮疹和ALT/AST(谷丙转氨酶/谷草转氨酶)水平升高^[65]^。这些安全性结果与过去其它ipilimumab临床研究一致^[65]^。Ipilimumab治疗黑色素瘤的临床经验显示，严重的致死性imARs可因药物的作用机制而增强；但是，若按照方案特定的治疗原则来处理，毒副反应通常是可控的，包括早期诊断标准以及使用高剂量糖皮质激素治疗可能影响胃肠道、皮肤、肝脏和内分泌系统的严重事件^[63, 67]^。患者早期报告症状，经培训的专科医生严格遵守指南^[63]^可降低危及生命的并发症风险^[67, 68]^。

在这些结果的基础上，计划进行一项随机、多中心、双盲、Ⅲ期临床研究(CA184-104)，旨在比较ipilimumab联合紫杉醇/卡铂与安慰剂联合紫杉醇/卡铂治疗Ⅳ期或复发鳞癌NSCLC的疗效。可采用ipilimumab的分阶段给药，即从第3化疗周期开始用药，合格的患者完成化疗后每12周接受一次维持治疗。主要目的是比较治疗组间的OS(clinicaltrials.gov编号NCT01285609)。

### Talactoferrin alfa

Talactoferrin alfa是一种重组人乳铁传递蛋白，因其糖基化方式不同而与由人乳汁中纯化得来的天然蛋白不同^[69]^。这种口服活性制剂具有免疫调节作用，可提高抗肿瘤细胞的先天免疫与适应性免疫^[70]^。例如，talactoferrin alfa诱导免疫细胞的运动，包括树突细胞，进入胃肠相关淋巴组织并在此刺激其成熟。这将导致携带肿瘤抗原的树突细胞含量增加。此外，talactoferrin alfa能够增加细胞因子的产生从而促进抗肿瘤CD8^+^T细胞和NK细胞的成熟与增殖。

在印度进行的2项晚期NSCLC随机Ⅱ期临床研究对talactoferrin alfa进行了评估。第一项临床研究中，110例既往一线或二线化疗失败的ⅢB/Ⅳ期患者，每日口服2次1.5 g talactoferrin alfa或安慰剂，持续12周，以14周为一个周期^[17, 71]^。与安慰剂相比，talactoferrin alfa明显改善中位OS(分别为6.0个月和3.7个月；HR=0.69；*P* < 0.05)并有延长PFS的趋势。Talactoferrin alfa耐受性良好，两组副反应和3/4度副反应发生率相近。

在第二项临床研究中，110例未经治疗的ⅢB/Ⅳ期NSCLC患者口服talactoferrin alfa或安慰剂联合紫杉醇/卡铂治疗^[72]^。Talactoferrin alfa剂量为1.5 g，每日2次，连续35天，在第1、3和5化疗周期后使用。在一线紫杉醇/卡铂治疗中加入talactoferrin有提高客观缓解率(分别为42%和27%；*P*=0.08)和延长中位OS的趋势(分别为10.4个月和8.5个月) ^[17, 72]^。Talactoferrin alfa的耐受性良好，与对照组相比副反应及3/4度副反应更少。

这些研究的大部分患者为印度裔，因此需要进一步确认这些发现是否适用于其他民族/种族。为回答这一问题，目前正在进行2项talactoferrin alfa Ⅲ期临床研究，称为FORTIS-M和FORTIS-C。FORTIS-M中，针对720例至少两次既往系统性抗癌治疗失败的ⅢB/Ⅳ期患者，比较安慰剂和talactoferrin alfa的疗效。OS是主要研究终点，预计2011年完成(clinicaltrials.gov编号NCT00707304)。FORTIS-C针对1, 100例晚期NSCLC患者，比较安慰剂与talactoferrin alfa联合紫杉醇/卡铂一线治疗的效果。OS和PFS作为共同的主要研究终点进行评估。预计2013年完成(clinicaltrials.gov编号NCT00706862)。根据Ⅱ期临床研究经验，两项研究中talactoferrin alfa的剂量为1.5 g，每日二次。

### Toll样受体9拮抗剂

Toll样受体(Toll-like receptors, TLRs)是识别病原相关分子模式的受体家族，调控抗原特异性的先天免疫。TLR9是该家族的一员，表达于树突细胞、T细胞、B细胞和类浆细胞样细胞。含非甲基化胞嘧啶-鸟嘌呤结构域的合成寡核苷酸可激活TLR9以降低免疫耐受性、促进肿瘤抗原识别与肿瘤细胞死亡^[16, 17, 73]^。

在一项Ⅱ期随机临床研究中，与每三周一次的一线紫杉醇/卡铂化疗联合，于第8天和第15天皮下注射0.2 mg/kg TLR9拮抗剂PF-3512676，表现有改善中位OS的趋势(分别为12.3个月和6.8个月，HR=0.747；*P*=0.188) ^[74]^。2项Ⅲ期国际临床研究已启动，评估分别与一线紫杉醇/卡铂化疗或吉西他滨/顺铂化疗联合的PF-3512676疗效；但是，中期分析提示与单纯化疗相比，增加PF-3512676并无获益，提前终止^[75, 76]^。其它TLR9拮抗剂，如IMO-2055，尚处于NSCLC早期研究中。

### 抗体依赖细胞介导的细胞毒性

抗体依赖细胞毒性(antibody-dependent cellular cytotoxicity, ADCC)是一种IgG1抗体与表达相应抗原的细胞结合并包被该细胞的过程，该过程募集NK细胞以介导抗体包被细胞的溶解^[77]^。目前若干可能使用ADCC的方法应用于多种肿瘤类型。一些证据表明，批准用于治疗头颈部鳞癌和结直肠癌的抗EGFR IgG1单克隆抗体西妥昔单抗，目前正在进行肺癌治疗的研究，至少部分是通过ADCC起作用的^[78, 79]^。值得注意的是，ADCC尚需在体内进行证实；所以，ADCC对临床肿瘤免疫原性的贡献程度尚不十分清楚。肿瘤患者，尤其是化疗患者中，存在免疫抑制环境，特征为T细胞和NK细胞在数量和功能上均有所降低，在该环境下ADCC活性可能会受影响^[80, 81]^。但是，进一步的临床前研究促使该想法成为一个命题^[78]^。一些临床前数据提示ADCC介导的和化疗介导的肿瘤细胞死亡甚至可能是免疫原性的，即它们“释放”肿瘤抗原提呈给T细胞，从而增强抗肿瘤的抗原特异性免疫应答^[82-85]^。所以，通过ADCC过程，西妥昔单抗之类的治疗可能能够激活免疫系统，尽管它们并无直接的免疫相关作用机制。

## 结论

尽管肺癌通常被认为不是一种免疫原性恶性肿瘤，但越来越多的证据表明提高抗肿瘤免疫应答在改善患者预后中举足轻重，包括治疗需求远未得到满足的患者的生存，如鳞癌和广泛期SCLC。有两种常用的免疫治疗方法，疫苗治疗旨在提高抗原刺激的免疫应答；非抗原免疫调控方法旨在降低肿瘤耐受性并提高激活的抗肿瘤免疫应答。

在Ⅱ期随机临床研究中，两种方法的疗效和安全性均达到了初始目的，正在进行的Ⅲ期试验旨在评估其能否改善目前标准治疗的相关疗效(表 3)。在这些Ⅲ期临床研究结果公布后，免疫治疗很可能整合入现有的治疗指南中，而不论肿瘤的组织学如何；可用于更多的肺癌人群而不论其分子标志物状态如何。

**3:** 目前正在进行的Ⅲ期NSCLC免疫试验总结

研究	治疗组	研究设计	计划入组患者数	主要终点	预计完成时间
NCT00409188	L-BLP25 *vs*.安慰剂	在不可切除的Ⅲ期NSCLC患者中进行的随机Ⅲ期研究	1, 476	OS	2011
NCT00415818	TG4010	在伴正常基线NK水平的MUC1+NSCLC患者中进行的Ⅱb/Ⅲ期研究	N/A	N/A	N/A
NCT00480025 (MAGRIT)	MAGE-A3 *vs*.安慰剂	在可切除的MAGE-A3+ⅠB, Ⅱ或ⅢA期NSCLC患者中进行的3期研究，按2:1随机分组(疫苗:对照)	2, 270	无病生存	2015
N/A	CimaVax EGF *vs*.安慰剂	在晚期(ⅢB/Ⅳ期)NSCLC患者中进行的Ⅲ期研究，按2:1随机分组(疫苗:对照)	579	在两个患者亚组中评估生存：年龄 > 60岁(*n*=381)和年龄≤60岁*n*=198)	N/A
NCT00676507	Belagenpumatucel-L *vs*.安慰剂	在Ⅲ/Ⅳ期NSCLC患者进行的随机Ⅲ期研究，一线化疗/化放疗后维持治疗	700	OS	2011
NCT01285609	Ipilimumab+紫杉醇/卡铂vs.紫杉醇/卡铂	在具鳞状细胞组织学特点的Ⅳ期或复发性NSCLC患者中进行的随机Ⅲ期研究	N/A	OS	N/A
NCT00707304 (FORTIS-M)	Talactoferrin alfa *vs*.安慰剂	在既往经≥2种全身抗晚期癌方案治疗失败的ⅢB/Ⅳ期NSCLC患者中进行的随机Ⅲ期研究	720	OS	2011
NCT00706862 (FORTIS-C)	Talactoferrin alfa *vs*.安慰剂，二者均联合一线紫杉醇/卡铂	在晚期NSCLC患者中进行的随机Ⅲ期研究	1, 100	OS和PFS	2013
EGF，表皮生长因子；MAGRIT，MAGE-A3辅助非小细胞肺癌免疫治疗；MUC1，粘液素1；N/A，不适用；NK，自然杀伤细胞；NSCLC，非小细胞肺癌；OS，总体生存期；PFS，无进展生存期。 注：本表得到版权所有者© International Association for the Study of Lung Cancer复制许可。					

为使肺癌患者免疫治疗的疗效最大化，确定哪些患者可从各种治疗中获益是至关重要的。鉴定生物标志物与进一步阐明这些治疗方法的作用机制有助于我们认识并帮助患者进行选择。

另一个重要的问题是免疫治疗的时机，免疫治疗是否与其他治疗联合，如化疗和放疗。在肿瘤发展得以逃逸免疫系统之前进行早期辅助治疗，免疫治疗的获益可能更多。尽管单纯的化疗会导致肺癌患者的预后较差。化疗与免疫治疗联合可产生协同作用。需要进行详细的研究来确定合适的剂量与方案，以获得最大疗效和最小毒性。

需要设计临床研究以可重复地评估免疫应答并研究其与预后的相关性。与化疗不同，免疫治疗时，免疫系统需要时间来激发对肿瘤的免疫应答，在评估抗肿瘤疗效前应将免疫应答考虑在内。尽管进行了大量的工作制定得到irRC以定义免疫治疗的特定疗效，这些标准在肺癌的研究中尚未进行充分的评估。需要进一步的工作来确定免疫制剂治疗肺癌的替代性研究终点。
